# Supplementary material for: Event-driven architecture and intelligent decision tree facilitated sustainable trade activity monitoring model design
Source: PLoS One. 2025 Sep 4;20(9):e0331663. doi: 10.1371/journal.pone.0331663 (PMC12410814; doi:10.1371/journal.pone.0331663)
Supplement: Attachment — Submitted filename: Response to Reviewers.docx [file pone.0331663.s001.docx]

Reviewer #1:

1. The description of the dp-OBDT algorithm lacks formal rigor. There are high-level explanations, but little is said about its computational complexity, convergence guarantees, or reproducibility.
In the main text, we conduct a detailed analysis of the algorithm and elaborate on the design principle of the algorithm as well as its computational complexity. The dp-OBDT algorithm performs an exhaustive search over all possible decision tree structures and is theoretically capable of identifying the global optimum under given constraints on depth and the number of feature nodes. However, due to the vastness of the search space, it may be impractical to explore all possibilities in practice. As a result, the algorithm’s convergence guarantee relies on the effectiveness of its search strategy and the efficiency of its caching mechanism.
Regarding reproducibility, the dp-OBDT algorithm is expected to produce consistent output given the same input data and parameter settings. Nevertheless, due to potential randomness in the search process—albeit significantly reduced by the caching mechanism—identical results may not always be guaranteed. That said, the overall trend and the quality of the optimal solution should remain consistent.

*The dp-OBDT (Dynamic Programming-based Optimal Binary Decision Tree) algorithm is designed to identify the optimal classification tree structure through an intelligent exhaustive search strategy. It models the decision-making process as a tree structure and leverages both historical and real-time data for analysis and prediction. During the search, the algorithm carefully designs traversal strategies to exploit the overlapping substructure of the tree and avoid unnecessary computation of suboptimal trees. This effectively keeps the time complexity of finding the optimal decision tree within a reasonable range. Specifically, the algorithm caches the boundary conditions of subtrees and the characteristics of optimal root nodes under constraints of feature depth and node count, thereby reducing redundant computations and improving search efficiency.*

*In terms of computational complexity, the primary overhead of the dp-OBDT algorithm arises from the exhaustive search over all possible decision tree structures. Although techniques such as caching and exploiting structural overlap help reduce the computational burden, the time complexity can still grow exponentially when applied to large-scale datasets. Nevertheless, compared to traditional optimal decision tree algorithms, dp-OBDT significantly reduces computation time through carefully designed traversal strategies and an efficient caching mechanism, enabling the discovery of near-optimal decision tree structures within a reasonable time frame.*

2. The model evaluation is based on a set of performance metrics (e.g., throughput, resource utilization, delay), but it’s not clear how these metrics are computed or whether the comparisons are statistically significant.

*Data backlog is measured by monitoring the number of unprocessed requests in the system. This is done by recording the length of the request queue at a given point in time. The size of the backlog reflects the system's processing capacity and real-time responsiveness—a larger backlog indicates slower processing and longer response times.
Resource utilization is calculated based on the average usage of system resources (e.g., CPU, memory) over a period of time. Higher utilization suggests more efficient use of available resources.
Throughput is estimated by calculating the number of requests successfully processed per unit of time (e.g., per second). We estimate this by combining the number of concurrent requests with the average response time to determine how many requests the system can handle per second.*

3. There are no measures of variance (e.g., standard deviation or confidence intervals).

The main objective of this paper is to demonstrate the effectiveness and advantages of the dp-OBDT algorithm in the monitoring of sustainable trade activities, rather than to conduct a detailed assessment of the statistical stability of the algorithm

4. It is unclear how many times the experiments were repeated. No statistical significance tests are provided. This makes it difficult to judge whether observed performance differences are robust or merely incidental. Include multiple runs for each experiment, report variance measures, and consider including t-tests or ANOVA for comparative metrics.

*To ensure the reliability and stability of the experimental results, each experiment was repeated 1,000 times, and the average was taken as the final result.* Although statistical significance tests are not included in this study, future work will consider incorporating methods such as t-tests or analysis of variance (ANOVA) to more accurately assess performance differences and algorithm robustness.

5. All underlying data are publicly available via recognized repositories such as Zenodo. The DOIs and URLs are properly provided in the Data Availability Statement.

*The Anneal dataset (*[*http://archive.ics.uci.edu/ml/index.php*](http://archive.ics.uci.edu/ml/index.php)*) is commonly employed for classification or regression tasks in machine learning and data mining. The UN Comtrade dataset (*[*https://comtrade.un.org/*](https://comtrade.un.org/)*) contains comprehensive merchandise trade statistics from official sources, covering over 97% of global trade in goods. The WDI dataset (*[*https://download.csdn.net/download/T0620514/88571060*](https://download.csdn.net/download/T0620514/88571060)*) includes data on various indicators such as GDP, GDP per capita, trade volume, investment, demographics, education, and health across multiple domains. The IMF dataset (*[*https://data.imf.org/en*](https://data.imf.org/en)*) provides a range of economic data, including balance of payments, foreign exchange reserves, exchange rates, and interest rates. The OECD dataset (*[*https://data.oecd.org/*](https://data.oecd.org/)*) offers information on import/export volumes, trading partners, commodity classifications, and other related areas. Based on these datasets, we present the parameter settings, where |D| represents the number of instances, |F| denotes the number of binary features, and |C| indicates the number of categories in the dataset.*

6. The manuscript is generally clear and well-organized. Each section flows logically into the next, and the technical descriptions are detailed. However, there are occasional grammatical issues and awkward phrasing that should be addressed during the copyediting stage. Examples include: Inconsistent use of technical vocabulary (e.g., "data accumulation" is ambiguous — does it refer to buffering, queuing, or something else?).

We gave the top-level meaning of data accumulation at order 4.2. Data accumulation reflects system delays by measuring the queue length of unprocessed requests at a given time—larger backlogs indicate slower processing and longer response times. And check the language description of the entire text to ensure that the language is concise and clear without ambiguity.

Reviewer #2:

1. Abstract should be more focused on novelty.

We reorganized the abstract to ensure that its language structure met the requirements.

*This paper introduces a groundbreaking monitoring model tailored for sustainable trade activity surveillance, which synergistically integrates event-driven architecture with an intelligent decision tree. Confronting the constraints of conventional trade monitoring approaches that falter in adapting to the intricate and ever-changing market landscape, our model innovatively establishes an efficient, adaptable, and sustainable monitoring framework. By embedding an intelligent decision tree, it enables dynamic resource allocation, thereby optimizing operational efficacy. Initially, we devise rules that align data injection and processing velocities, ensuring expedient data processing. Subsequently, we implement an optimal binary tree decision-making algorithm, grounded in dynamic programming, to achieve precise allocation of elastic resources within data streams, significantly bolstering resource utilization. Throughout the monitoring continuum, the model employs intelligent agents to assess resource status in real-time and dynamically adjusts resource allocation strategies triggered by events, prioritizing the seamless execution of pivotal trade activities. Empirical findings underscore the model's superiority across critical metrics, including data accumulation efficiency, processing latency, resource utilization, and throughput. Specifically, it attains an average data accumulation value of 15.46, curtails latency by 14.67%, achieves an average resource utilization of 60.29%, and registers a throughput of 336.5 Mbps. Consequently, the model not only exhibits rapid responsiveness to market fluctuations and curtails resource energy consumption but also fosters a harmonious equilibrium between economic gains and environmental preservation, ensuring the uninterrupted operation of trade activities.*

2. Introduction does not highlight the motivations of the study. Highlight the motivations of the study in the introduction.

*Although some studies have attempted to apply emerging technologies to trade activity monitoring, existing approaches remain inadequate in coping with the complexity and dynamism of modern market environments. In particular, traditional monitoring models struggle with stream processing applications involving multi-source inputs and multi-directional outputs due to rigid architectural designs and limited scalability. This often leads to mismatches between data ingestion and processing rates, resulting in increased processing delays and inefficient resource utilization. Such limitations not only reduce the effectiveness of trade monitoring but also hinder the achievement of dual goals: economic efficiency and environmental sustainability. Therefore, there is an urgent need for a new monitoring model capable of real-time market sensing, adaptive resource allocation, and intelligent decision-making to overcome the constraints of traditional methods and enhance the overall sustainability of trade activities.*

3. In Section 2 (related work), citing references is not appropriate. For example, literature [11], etc. Author's names should be included instead of word "literature".

We have modified the reference format of the citation.

4. I did not find figure 1 in the article except for its caption.

We have revised the pictures in the thesis to ensure the integrity of the thesis structure.

5. Equation 3, what us w? I did not see any "w" before.

w is the processing interval w of the event, which we explained when introducing Formula 1.

6. Equation writing can be improved. e.g., Eqn 4.

We inspected and improved the format of the formula.

7. Check all the figures because they are not visible in the article.

We modified and completed the missing pictures in the original file to ensure the integrity of the data.

8. Can you provide the references for Edn 8? Is there only one method to check the precision?

We have provided the source of Formula 8, which is an authoritative formula setting in the industry

Bishop C M, Nasrabadi N M. Pattern recognition and machine learning[M]. New York: springer, 2006.

10. Discussion is not enough to explain the results. Your results are good to discuss. Enhance the discussion.

*A deeper analysis of the experimental results presented in Sections 4.3 to 4.5 demonstrates that the proposed model exhibits significant advantages in runtime efficiency, providing a solid foundation for real-time monitoring and instant feedback in trade activities. The experiments show that the substantial reduction in execution time enables the system to promptly respond to various events in trade scenarios. When early signs of potential environmental risks—such as excessive carbon emissions—emerge, the system can quickly identify these issues and trigger appropriate response mechanisms to contain negative impacts in a timely manner. This capability is crucial for supporting the green and sustainable development of trade operations.*

*Moreover, the shorter runtime enhances the decision-making speed of the intelligent decision tree, offering timely and reliable guidance for adaptive adjustments and optimizations in trade activities. This accelerates the transition of trade processes toward a sustainable development path.*

*From the perspective of throughput, the high-throughput characteristics of the system allow for the full utilization of computing resources, avoiding idleness and waste, and thereby promoting carbon emission reductions at the resource usage level. The observed reduction in data backlog during experiments not only decreases data processing latency and improves system responsiveness but also frees up storage capacity, optimizing storage resource allocation and minimizing emissions associated with data storage. Additionally, high-quality data inputs enhance the accuracy of the intelligent decision tree, resulting in decisions that better reflect actual demand and resource conditions. This enables dynamic optimization of resource allocation, ensuring that while maintaining the normal operation of trade activities, the system can precisely reduce unnecessary resource consumption. Ultimately, this leads to lower carbon emissions and supports energy conservation and emission reduction goals, offering comprehensive technical support for the sustainable development of trade.*

11. Conclusion is general. Revise it and focus more on the summary of the results.

*This paper proposes a novel monitoring model for sustainable trade activities by integrating an event-driven architecture with an intelligent decision tree. Methodologically, the model ensures data consistency, reserves computing resources through dynamic programming for optimal binary decision tree construction, and employs intelligent agents for dynamic decision-making, enhancing adaptability to complex trade environments. The intelligent decision tree builds a fine-grained model that comprehensively considers multiple factors to provide scientifically grounded resource allocation strategies. Under the event-driven architecture, the model can respond rapidly to events, flexibly adjust resource distribution, prioritize critical trade activities, and improve overall efficiency and sustainability.*

*Experimental results confirm the effectiveness of the proposed model. It demonstrates high monitoring accuracy, capable of capturing critical information with precision. Its decision-making process is transparent and traceable, with strong credibility and trustworthiness that foster user confidence. The model also exhibits strong stability, ensuring reliable and continuous system operation. Overall, it offers an efficient, intelligent, and dependable solution for monitoring sustainable trade activities.*

*Future work will focus on algorithmic optimization to improve the adaptability and accuracy of the intelligent decision tree in more complex trade scenarios. Additionally, efforts will be made to incorporate more real-time data sources and event types, enhancing the model’s situational awareness and responsiveness. By integrating machine learning and artificial intelligence technologies, the model aims to achieve smarter resource allocation and decision support, contributing to the long-term development of sustainable trade activities.*
