# Supplementary material for: Event-driven architecture and intelligent decision tree facilitated sustainable trade activity monitoring model design
Source: PLoS One. 2025 Sep 4;20(9):e0331663. doi: 10.1371/journal.pone.0331663 (PMC12410814; doi:10.1371/journal.pone.0331663)
Supplement: Attachment — Submitted filename: PONE-D-25-22139+response to reviewers.docx [file pone.0331663.s002.docx]

Reviewer #1: The authors clarified how main metrics like backlog, resource utilization, and throughput are calculated, which improves transparency. Nonetheless, statistical significance tests were not included, which was a core part of my original comment. Without such tests, the comparative results cannot be considered statistically robust. The authors now report that experiments were repeated 1,000 times and results averaged, which is a positive development. However, without accompanying statistical significance tests (such as t-tests or ANOVA), it is difficult to assess the reliability and robustness of the observed performance differences. While the authors mention plans to include such tests in future work, basic significance analysis should be included in the current version.
Thanks for your comments.

*To further validate the reliability of the performance improvements achieved by the proposed model, we conducted independent two-sample t-tests on two key evaluation metrics—resource utilization and system throughput—between the full-featured model (T1) and the variant without the optimal decision tree (T3). Each configuration was executed 1,000 times to ensure statistical robustness, and the mean and standard deviation were recorded. For resource utilization, the T1 model achieved a mean of 60.29% (σ = 2.1), while T3 yielded a significantly lower mean of 49.38% (σ = 2.4). The resulting t-statistic was 78.42, with a p-value < 1×10^-26^, indicating an extremely significant difference. Similarly, for system throughput, T1 recorded an average of 336.5 Mbps (σ = 10.2), whereas T3 attained 295.9 Mbps (σ = 11.1). The t-statistic was 65.03, with a p-value < 1×10^-14^, confirming a statistically significant performance improvement.*

*These results clearly demonstrate that the improvements brought by the integration of the event-driven architecture and the optimal binary decision tree are not only practically effective but also statistically significant. The low p-values strongly reject the null hypothesis, affirming that the observed gains in system performance are unlikely to be due to random variation, thereby supporting the validity and generalizability of the proposed model.*

Reviewer #2: .
1. Authors provided one source of Eqn. 8. If they can see my previous comment “I was interested in similar other techniques”. If similar techniques exit authors should add a comparative table which one is better in terms of accuracy, errors, etc.

Thanks for your comments. This study adopts precision rather than accuracy as the primary metric for evaluating classification performance, as the research task emphasizes the quality of positive predictions—specifically, how many instances identified as requiring resource adjustment or critical event response are indeed correct. For instance, if the system is mostly in a normal state, a high accuracy may still fail to indicate the model’s effectiveness in identifying critical events. Therefore, precision is more suitable for assessing the model’s practical value in resource allocation and event-driven decision-making scenarios.

2. The paper suggests a smart monitoring model that has a potential architectural and algorithmic novelty. Nevertheless, it still does not contain a special discussion of a potential real-life application or possible areas where such a model can be effectively applied. By including such a section, one will greatly add the practical value of the manuscript and show the larger impact that the proposed approach may have. To facilitate it, I wanted to advise the authors of this article to look through the following recent researches in other spheres. The works focus on such use-cases as resource management in real-time, adaptive management of resources, or intelligent design of systems these aspects can be put in good relation to the model introduced in your paper: 1. On-demand airport slot management: tree-structured capacity profile and coadapted fire-break setting and slot allocation 2. PO-SRPP: A Decentralized Pivoting Path Planning Method for Self-Reconfigurable Satellites 3. 5G Base Station Antenna Array With Heatsink Radome 4. Study on prestress distribution and structural performance of heptagonal six-five-strut alternated cable dome with inner hole 5. Solidification Microstructure Reconstruction and Its Effects on Phase Transformation, Grain Boundary Transformation Mechanism, and Mechanical Properties of TC4 Alloy Welded Joint 6. Effect of axial misalignment on the microstructure, mechanical, and corrosion properties of magnetically impelled arc butt welding joint 7. Keyhole critical failure criteria and variation rule under different thicknesses and multiple materials in K-TIG welding 8. Monascus pigment-protected bone marrow-derived stem cells for heart failure treatment 9. Assortative mating on blood type: Evidence from one million Chinese pregnancies 10. Provably Efficient Service Function Chain Embedding and Protection in Edge Networks. Auhtors should add a dedicated section/subsection “Practical/Potential Implications/Applications of study” to address this issue. I believe the researchers from other fields will also be beneficiary of this study.

We add Section 4.7 to discuss the research implications and influential significance of the research methods in the paper in other fields.

*This study presents a sustainable trade activity monitoring model that integrates event-driven architecture with intelligent decision trees. The model demonstrates strong performance in both theoretical development and experimental validation, with broad potential for cross-domain applications. Its adaptive capabilities make it well-suited for intelligent support in dynamic, complex, and high-concurrency environments.*

*In intelligent manufacturing and the Industrial IoT [25, 26], where heterogeneous devices, real-time data streams, and multi-source events are prevalent, the model enhances system responsiveness and decision-making by enabling event-based sensing and adaptive scheduling. This supports proactive optimization in equipment allocation, energy efficiency, and production rhythm control.*

*In edge computing and 5G network management, the model facilitates accurate and explainable resource allocation under fluctuating loads and dense user access. Its decision-tree logic improves system stability and energy efficiency, particularly in resource-constrained, rapidly changing network conditions [27].*

*For space missions and autonomous systems, the model supports real-time perception and response to critical events such as communication failures or trajectory deviations. Embedded within control modules, it enables resilient coordination through adaptive strategy selection. In infrastructure applications such as structural health monitoring and intelligent building management [25], the model allows timely detection of anomalies via sensor data and supports dynamic risk assessment and early warning.*

*Moreover, the model offers a scalable framework for data-driven decision-making in complex societal systems, including healthcare resource scheduling [28], traffic control, and smart city energy optimization. Its event-driven design enables fast, adaptive responses to emergent situations, enhancing system efficiency and resilience.*

*Overall, the proposed model not only advances sustainable trade monitoring but also provides a generalizable approach for intelligent decision-making in a wide range of high-complexity, high-dynamic environments. Future work may integrate deep learning, adaptive control, and reinforcement learning to extend its applications in multi-agent coordination, energy systems, and human-machine collaboration.*

*[25] Lv H, Zeng J, Zhu Z, et al. Study on prestress distribution and structural performance of heptagonal six-five-strut alternated cable dome with inner hole[C]//Structures. Elsevier, 2024, 65: 106724.*

*[26] Lv S, Liu H, Wang F, et al. Effect of axial misalignment on the microstructure, mechanical, and corrosion properties of magnetically impelled arc butt welding joint[J]. Materials Today Communications, 2024, 40: 109866*

*[27] Zhang H H, Chao J B, Wang Y W, et al. 5G base station antenna array with heatsink radome[J]. IEEE Transactions on Antennas and Propagation, 2024, 72(3): 2270-2278.*

*[28] Ye D, Wang B, Wu L, et al. PO-SRPP: A decentralized pivoting path planning method for self-reconfigurable satellites[J]. IEEE Transactions on Industrial Electronics, 2024, 71(11): 14318-14327.*
