# Supplementary material for: Event-driven architecture and intelligent decision tree facilitated sustainable trade activity monitoring model design
Source: PLoS One. 2025 Sep 4;20(9):e0331663. doi: 10.1371/journal.pone.0331663 (PMC12410814; doi:10.1371/journal.pone.0331663)
Supplement: Attachment — Submitted filename: PONE-D-25-22139+response_to_reviewers_auresp_3.docx [file pone.0331663.s003.docx]

Reviewer #2: If you can see Comment 2, I suggested adding the possible implications in other fields. Authors added very limited fields. Authors must improve the newly added section. It will enhance the readability and the applicability of the study for the experts of other fields as well.

Response: We sincerely thank the reviewer for this insightful comment. We fully agree that a broader discussion of the potential implications in other fields would strengthen the manuscript and enhance its value for experts beyond the trade monitoring domain. In the revised version, we have substantially expanded the Discussion section.

“the proposed monitoring model demonstrates substantial potential for effective deployment in real-world domains. For instance, the dynamic event-driven allocation strategy aligns closely with challenges in airport slot management, where tree-structured capacity planning and adaptive allocation mechanisms can substantially reduce congestion and enhance operational efficiency (Wang et al., 2024). The parallel between air traffic flow and trade activity streams highlights the universal importance of synchronizing resource injection and processing rates. Just as the proposed model achieves consistency between upstream and downstream applications, airport slot management requires a balance between slot allocation and real-time passenger or cargo flow. The ability of the model to dynamically adjust computational resources mirrors the adaptive allocation of limited airport capacity, thereby ensuring system resilience under fluctuating demand conditions.

Similarly, the decentralized decision-making capability of the model resonates with autonomous satellite management, particularly in the context of pivoting path planning and reconfigurable satellite coordination (Ye et al., 2024). In this domain, satellites must autonomously reconfigure their trajectories to adapt to unforeseen environmental changes, resource limitations, or mission requirements. The event-driven and decision-tree-based logic embedded in the proposed monitoring framework offers a blueprint for such adaptive responses. Specifically, the model’s intelligent agent–driven allocation mechanism can be extended to satellite clusters, enabling real-time coordination and distributed optimization in highly dynamic environments. This analogy underscores the generalizability of the model across systems characterized by resource constraints, event-driven triggers, and high concurrency requirements.”
